# Association of Oxytocin and Parental Prefrontal Activation during Reunion with Infant: A Functional Near-Infrared Spectroscopy Study

**DOI:** 10.3389/fped.2017.00271

**Published:** 2017-12-18

**Authors:** Jun Ito, Takeo Fujiwara, Yukifumi Monden, Takanori Yamagata, Hideki Ohira

**Affiliations:** ^1^Department of Pediatrics, Yokohama City Minato Red Cross Hospital, Yokohama, Japan; ^2^Department of Global Health Promotion, Tokyo Medical and Dental University, Tokyo, Japan; ^3^Department of Social Medicine, National Research Institute for Child Health and Development, Tokyo, Japan; ^4^Department of Pediatrics, Jichi Medical University, Shimotsuke, Japan; ^5^Department of Psychology, Nagoya University, Nagoya, Japan

**Keywords:** functional near-infrared spectroscopy, oxytocin, parenting, holding, lateralization

## Abstract

Although previous studies have revealed the role of oxytocin (OT) in parental behavior, the role of OT has not been investigated through the direct assessment of prefrontal brain activation during parenting. By using functional near-infrared spectroscopy, we aimed to show the relationship between parental [maternal (*N* = 15) and paternal (*N* = 21)] OT levels and the activation of the prefrontal cortex (PFC), while holding their infants after separation. Baseline OT levels were measured in the subjects’ saliva samples before the experiment. Prefrontal brain activation was assessed in participants sitting alone on a chair (i.e., separation from their infant for 120 s) and during the target period (i.e., holding their infant for 45 s), which was done in triplicate. The oxygen hemoglobin (oxy-Hb) dissociation curve significantly increased in 9 out of 22 channels on the PFC when maternal and paternal samples were combined. However, only the fathers showed a correlation between salivary OT and oxy-Hb signal. Furthermore, while holding their infants, high-OT fathers showed left hemispheric dominance compared to low-OT fathers, while high-OT mothers showed right hemispheric dominance compared to low-OT mothers. This study showed that fathers with high-OT levels showed neural activation with left hemispheric dominance, while holding their infants, suggesting that increase of OT level might activate paternal PFC related to parenting behavior, although the same is not true for mothers.

## Introduction

Brain activity of parenting is composed of a complex array of factors, such as attention to infant signals, evaluating infant condition, or decision of parental behavior. Because adequate parenting is essential not only for the survival of human infants, but also for their cognitive and emotional development, understanding the brain basis of early parent–infant interaction is worthy a challenge (1). A number of previous studies suggest the association between neuroendocrine and parenting (1, 2). The nine amino-acid neuropeptide, oxytocin (OT), plays a role in parenting both animal and human studies (2). Administration of OT enhances parenting behavior in rats (3), sheep (4), and humans (5), whereas, baseline plasma OT levels are related to normal parental behavior both in mothers and fathers (6, 7).

Oxytocin, a neurotransmitter (8), is considered to affect brain activity during parenting (9). The association between OT and parenting could, therefore be explained by analyzing brain activity in targeted regions. For example, Febo et al. confirmed that OT administration activates certain brain regions, including the prefrontal cortex (PFC), using functional magnetic resonance imaging (fMRI) in rats (10). Strathearn et al. used fMRI in human mothers who viewed pictures of their infants’ faces. This activated the ventral striatum and hypothalamus and correlated with plasma OT levels (11). Atzil et al., revealed similar correlations between maternal plasma OT and the activation of the left nucleus accumbens (NAcc) and the right amygdala, while they watched videotaped mother–infant interaction (12).

However, these studies relied on pictures or videos of their own infants to stimulate parenting-based brain activation, which is limited to auditory and visual stimulation and may not reflect a holistic scenario that includes olfactory and tactile sensations (13–16). Touch is particularly important and forms the basis of parent–infant relationships; both touching behavior from parent to infant, and interaction with the infant are associated with increased parental OT levels (17). The ideal experiment would, therefore be to detect brain activation while parents interact with their infants directly.

For more than a decade, non-invasive functional near-infrared spectroscopy (fNIRS) has been used to detect the hemodynamic response of cerebral cortices indicating cerebral activation. The fNIRS provides increased mobility compared to fMRI, and is more suitable to use in real settings (18). Therefore, it was anticipated that it would be possible to detect parental brain activation using fNIRS, while seated subjects were holding their infants.

As one of the cortices that can be visualized by fNIRS during activation, we focused on the PFC for two reasons. First, the orbitofrontal cortex (OFC) was recently shown to be a part of a neural network of parenting in the context of social recognition and reward systems. That is, the OFC is a potential region for coordinating the link between social signals (such as reunion with infant) and affective information (such as affection activated through rewarding system) (1). Second, previous fMRI studies of mothers receiving visual stimulation of their own infants showed significant activation of the dorsolateral and medial PFC (11) or the medial and superior frontal gyri (12).

By showing the association between OT and prefrontal brain activity, we may elucidate the mechanism of the association between OT and parenting. This evidence would add the rationale of investigating the impact of increase of OT level for appropriate parenting, using direct method [e.g., nasal spray of OT (19)] or indirect method [e.g., touching or hugging (20)].

In summary, by using fNIRS, we aimed to verify the hypothesis that PFC activation observed in parents while holding their infants, compared to baseline activity during separation, is associated with OT levels. Further, we investigated the association stratified by maternal and paternal subjects. Through this experiment, the brain basis of parent–infant interaction would be revealed in more dynamic and real settings than previous studies, and the results might be applied for parents who have difficulties of parenting.

## Materials and Methods

### Participants

In the framework of a Birth Cohort Study in National Center for Child Health and Development (NCCHD), 28 couples were recruited during their 3-month infant health-care visit at the NCCHD in Japan. At the NCCHD, located in a western suburb of Tokyo, 1,500–1,800 babies are delivered annually. Pregnant women were recruited between December 2010 and February 2013 (*N* = 2,405). Among them, 28 couples (*N* = 56) visited our laboratory when their infants reached the age of 4–6 months. Mothers taking prescription drugs for depression and anxiety (*N* = 1), and left-handedness (*N* = 1) [assessed by the Edinburgh Handedness Inventory (21)] were excluded. Due to insufficient saliva samples (*N* = 5) and fNIRS artifacts (*N* = 13), the final analysis included 15 mothers [age range 28–48, average 38.2 (SD = 5.77)] and 21 fathers [age range 27–54, average 39.0 (SD = 6.85)].

This study was approved by the ethics committee of the NCCHD, and all participants signed informed consent forms.

### OT Analysis

All participants arrived at the laboratory between 10 a.m. and 3 p.m. Parents were asked to refrain from food intake and nursing, 30 min before arriving. Infants were separated from their parents at least 15 min before sample collection. Saliva samples were collected using a Salivette Saliva Collection Device (Sarstedt, Rommelsdorft, Germany). Parents were asked to chew a roll of cotton until it was saturated. Salivettes were kept ice-chilled before being centrifuged at 4°C at 1,500 × g for 15 min and the liquid samples stored at −80°C. To concentrate the samples by threefold or fourfold, the liquid samples were lyophilized overnight and kept at −20°C until assayed. The dry samples were reconstructed in the assay buffer immediately before analysis using an OT EIA commercial kit (Assay-Design, Ann Arbor, MI, USA), consistent with previous research (22).

### fNIRS Recording Procedure

Using the multichannel fNIRS system (ETG-4000, Hitachi Medical Co,. Japan), hemodynamic changes were measured in specific PFC regions. The instrument generates two wavelengths of near-infrared light (695 and 830 nm) and measures the levels of oxy-Hb, deoxyhemoglobin (deoxy-Hb), and their sum continuously. NIRS probes were arranged in a 3 × 5 rectangular lattice resulting in 22 channels, and placed on the subject’s forehead in order to cover the prefrontal cortices. The lowest lines of the probes were positioned in a direction parallel to the T3-Fp1-Fp2-T4 line in the international 10–20 system.

**Figure 1:**
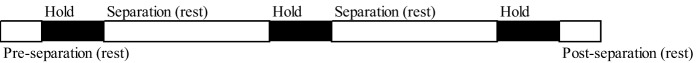
Procedure of separation of infant and parent.

Each subject was seated on a chair in a quiet room and separated from their infant and partner when the fNIRS recording started. The chair was positioned in front of the door, so that the infant could easily be transferred to the parent. The infant was passed between the subject and partner across the door following the instruction from staff. Staff helped in opening and closing the door. The subject was allowed to hold and touch the infant, but not allowed to move suddenly or stand up from the chair. The duration of reunion, which is the target block of fNIRS recording, was approximately 45 s and repeated three times. The first two target blocks were followed by 120 s separation periods as washout intervals for each block. The baseline block was defined as the 10 s just before the first target block and after the final block (Figure 1).

Oxygen hemoglobin signals were used for analysis due to its higher signal amplitude than that of deoxy-Hb (23). Individual timeline data for the oxy-Hb signals of each channel were preprocessed with a first-degree polynomial fitting and high-pass filter using cutoff frequencies of 0.003 Hz to remove baseline drift, and a 0.8 Hz low-pass filter to remove heartbeat pulsations. From the preprocessed time series data, we obtained channel-wise and subject-wise contrasts by calculating the inter-block means, and differences between the target and baseline periods.

### fNIRS Channels Spatial Estimation

For spatial profiling of fNIRS data, we employed virtual registration to register fNIRS data to the Montreal Neurological Institute (MNI) brain space (24, 25). Briefly, this method allows one to place a virtual probe holder on the scalp by simulating the holder’s deformation and by registering probes and channels onto reference brains in an MRI database (26, 27). We performed a statistical analysis of the MNI coordinate values for the fNIRS channels to obtain the most likely estimate of the location of given channels for subjects, and the spatial variability associated with the estimation (28). Finally, the estimated locations were anatomically labeled using a Matlab function that reads anatomical labeling information coded in both a macroanatomical brain atlas (LBPA40) (29) and Brodmann’s atlas (30) are shown in (Table 1).

**Table 1:** Spatial profiles of the channels among region-of-interest (ROI).

MNI coordinates *x*, *y*, *z* (SD)		Macroanatomy	Prob	Brodmann area	Prob
Ch1	−35, 63, −4 (4)	L middle frontal gyrus (MHG)	0.9	10, Frontopolar area	0.91
		L inferior frontal gyrus (IFG)	0.1	11, Orbitofrontal area	0.09
Ch4	38, 63, −4 (4)	R IFG	0.78	10, Frontopolar area	1
		R MHG	0.22		
Ch5	−44, 53, 6 (6)	L IFG	0.81	46, Dorsolateral prefrontal cortex (DLPFC)	1
		L MHG	0.19		
Ch6	−24, 68, 9 (5)	L MHG	1	10, Frontopolar area	0.99
				11, Orbitofrontal area	0.01
Ch7	2, 69, 8 (7)	R superior frontal gyrus (SFG)	0.64	10, Frontopolar area	1
		L SFG	0.36		
Ch8	27, 68, 9 (5)	R MHG	1	10, Frontopolar area	0.99
				11, Orbitofrontal area	0.01
Ch9	47, 52, 7 (6)	R IFG	0.94	46, DLPFC	1
		R MHG	0.06		
Ch10	−35, 58, 20 (6)	L MHG	1	46, DLPFC	0.98
				10, Frontopolar area	0.02
Ch13	37, 57, 20 (6)	R MHG	1	46, DLPFC	0.89
				10, Frontopolar area	0.11

### Analyses

Statistical analyses were performed in a channel-wise manner on oxy-Hb signals. To screen the channels involved in reunion with infants, target vs. baseline contrasts were subjected to a one-sample *t*-test against zero (two-tails). A statistical threshold was set at 0.05 with the Bonferroni method. The channels with significant activation were subjected to correlation analyses between salivary OT and oxy-Hb signals.

For the analyses of the hemispheric dominance for parenting, the laterality indexes (LI) of the pair of fNIRS channels among the region-of-interest (ROI) in a symmetrical position (Ch1–4, Ch5–9, Ch6–8, Ch10–13) and the LI of mean signal change among the left four channels (Ch L; Ch1, 5, 6, 10) and the right channels (Ch R; Ch4, 8, 9, 13) were calculated following the classic formula used for MRI studies (31):

LI=(QL−QR)/(QL+QR).

The positive LI means left hemispheric dominance of the brain, and *vice versa*. Then a two-way factorial ANOVA was conducted on data from mothers and fathers according to the salivary OT level (high and low), and the simple main effects were calculated.

## Results

The results of salivary OT levels are shown in Table 2. Both mothers and fathers were divided into high- and low-OT by median, respectively. No statistical differences were observed between mothers and fathers, for total, high- and low-OT groups (all *p* > 0.2).

**Table 2:** Salivary OT levels among mothers and fathers.

	Mothers			Fathers				
	*n*	Mean (SD) (pg/mL)	Median (range)	*n*	Mean (SD) (pg/mL)	Median (range)	*t*	*p*
Total	15	30.42 (14.9)	26.36 (15.67–73.39)	21	32.62 (16.6)	29.14 (10.70–79.41)	−0.41	0.69
Low-OT	8	22.56 (3.75)	22.84 (15.67–26.36)	10	19.55 (5.37)	18.50 (10.70–27.61)	1.34	0.20
High-OT	7	39.41 (6.78)	30.78 (26.97–73.39)	11	44.51 (14.0)	42.49 (29.14–79.41)	−0.89	0.39

*t-values and p-values were the results of t-tests between mothers and fathers*.

*OT, oxytocin; t, t-value; p, p-value*.

**Figure 2:**
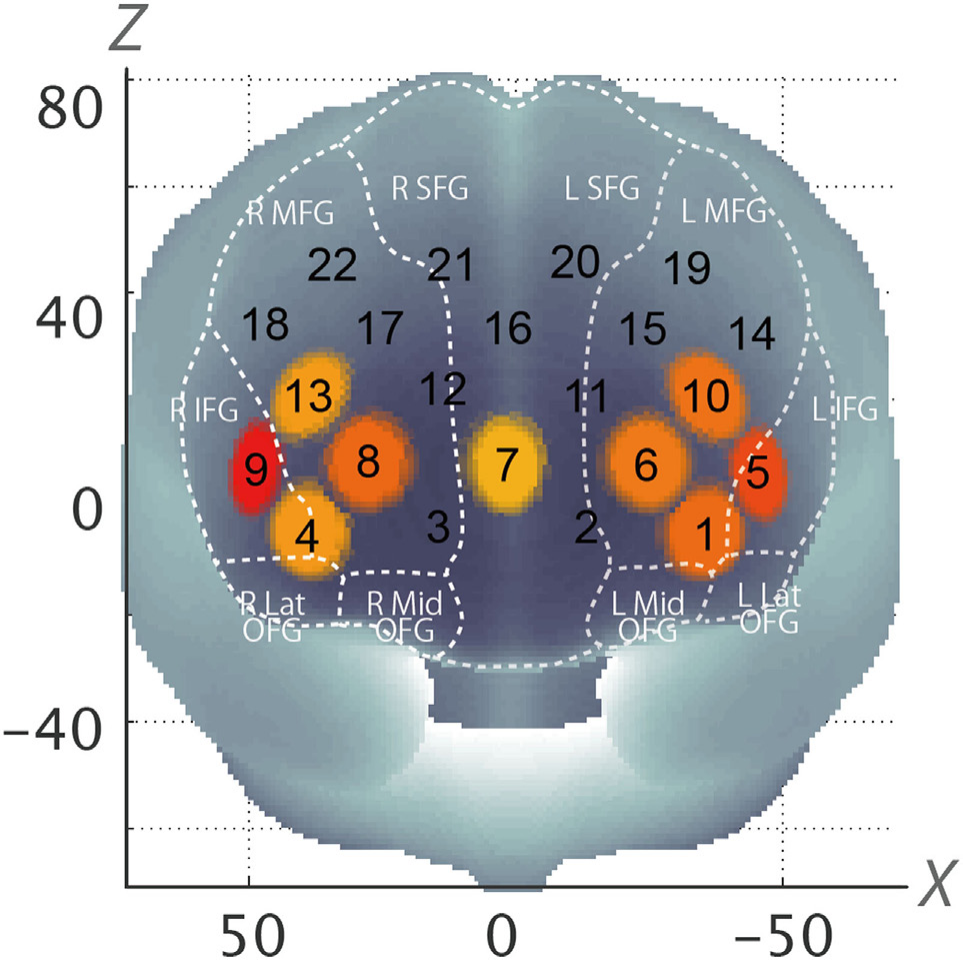
Statistically estimated fNIRS channel locations for the subjects are exhibited in MNI space. Channels showing significant oxy-Hb change are circled and colored yellow to red depending on *p*-values (Corresponding to Table 3). SFG, superior frontal gyrus; MFG, middle frontal gyrus; IFG, inferior frontal gyrus; OFG, orbitofrontal gyrus; oxy-Hb, oxygen hemoglobin; fNIRS, functional near-infrared spectroscopy; MNI, Montreal Neurological Institute.

In the whole brain analysis, using all samples, significant oxy-Hb increases were observed in nine channels (Ch1, Ch4–10, Ch13) symmetrically on the prefrontal area, which includes the OFC, the dorsolateral prefrontal cortex (DLPFC), and the frontopolar cortex (FPC) when parents, both mothers and fathers, reunited with their infants (Table 3; Figure 2). Those channels were selected as ROI for the following analyses. The change of oxy-Hb signals of three channels (Ch1, Ch7, and Ch8) in the ROI was correlated with the salivary OT level.

**Table 3:** Functional data summary for all subjects and correlations between oxy-Hb signal change and salivary OT levels.

	Oxy-Hb signal change				Correlations
Channel	Mean	SD	*t*	*p*	*r*
Ch1	0.128	0.161	4.76	**0.001**	**0.39**
Ch2	0.076	0.169	2.72	0.223	0.27
Ch3	0.111	0.212	3.14	0.075	**0.36**
Ch4	0.110	0.179	3.69	**0.017**	0.25
Ch5	0.197	0.220	5.37	**0.000**	0.18
Ch6	0.114	0.156	4.38	**0.002**	0.31
Ch7	0.106	0.186	3.41	**0.037**	**0.44**
Ch8	0.146	0.180	4.86	**0.001**	**0.35**
Ch9	0.162	0.152	6.40	**0.000**	0.27
Ch10	0.133	0.174	4.61	**0.001**	0.21
Ch11	0.071	0.154	2.76	0.201	**0.43**
Ch12	0.073	0.148	2.95	0.125	**0.41**
Ch13	0.102	0.164	3.73	**0.015**	0.13
Ch14	0.082	0.267	1.85	1.615	0.23
Ch15	0.004	0.138	0.19	18.720	**0.40**
Ch16	0.024	0.157	0.93	7.899	0.23
Ch17	0.043	0.133	1.93	1.346	0.26
Ch18	0.076	0.169	2.70	0.232	−0.10
Ch19	−0.005	0.169	−0.18	18.886	0.26
Ch20	0.034	0.141	1.46	3.358	0.29
Ch21	0.030	0.135	1.33	4.241	0.13
Ch22	0.016	0.143	0.65	11.418	-0.04

*Oxy-Hb values are presented in units of mM⋅mm*.

*OT, oxytocin; t, t-value; p, p-value; r, correlation coefficient; oxy-Hb, oxygen hemoglobin*.

*Bold values signifies p < 0.05*.

When stratified by gender, among fathers, all of the channels in the ROI (Ch1, Ch4–10, Ch13) showed significant increases in the oxy-Hb signal (all *p* < 0.05). However, among mothers, three channels in the ROI were not significantly affected (Ch4, Ch7, and Ch13). Furthermore, the significant correlation between the oxy-Hb signal changes and salivary OT levels were only seen among fathers at Ch1 and Ch7 (Table 4).

**Table 4:** Functional data summary and correlations between oxy-Hb signal change and salivary OT levels for ROI channels among mothers and fathers, respectively.

	Mothers (*n* = 15)					Fathers (*n* = 21)				
	Oxy-Hb signal change				Correlations	Oxy-Hb signal change				Correlations
Channel	Mean	SD	*t*	*p*	*r*	Mean	SD	*t*	*p*	*r*
Ch1	0.089	0.123	2.81	**0.014**	0.24	0.156	0.182	3.92	**0.001**	**0.45**
Ch4	0.085	0.205	1.60	0.131	0.16	0.128	0.160	3.65	**0.002**	0.32
Ch5	0.183	0.186	3.80	**0.002**	−0.23	0.207	0.246	3.86	**0.001**	0.36
Ch6	0.121	0.099	4.76	**0.000**	0.06	0.109	0.189	2.64	**0.016**	0.41
Ch7	0.051	0.100	1.95	0.071	0.16	0.145	0.223	2.98	**0.007**	**0.54**
Ch8	0.115	0.131	3.41	**0.004**	0.18	0.168	0.209	3.68	**0.001**	0.42
Ch9	0.139	0.105	5.14	**0.000**	0.16	0.179	0.179	4.57	**0.000**	0.30
Ch10	0.144	0.154	3.63	**0.003**	−0.06	0.126	0.190	3.03	**0.007**	0.35
Ch13	0.050	0.103	1.86	0.084	−0.28	0.140	0.190	3.36	**0.003**	0.25

*Oxy-Hb values are presented in units of mM⋅mm*.

*OT, oxytocin; SD, standard deviation; t, t-value; p, p-value; r, correlation coefficient; oxy-Hb, oxygen hemoglobin; ROI, region-of-interest*.

*Bold values signifies p < 0.05*.

**Figure 3:**
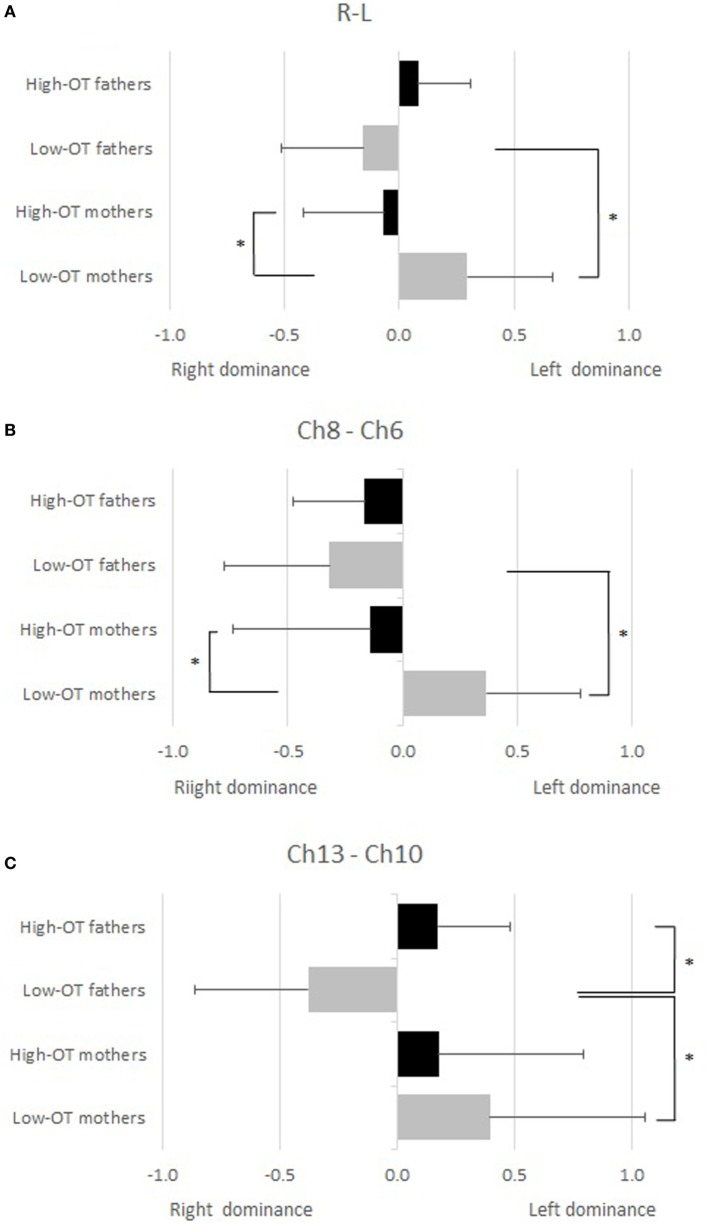
A two-way factorial ANOVA was conducted for the laterality indexes (LI) of the pair of functional near-infrared spectroscopy channels to mothers and fathers according to the salivary oxytocin (OT) level (high and low). Significant conditions (simple main effect, *p* < 0.05) are indicated by asterisks. **(A)** Mean signal change among the left four channels (Ch L; Ch1, 5, 6, 10) and the right four channels (Ch R; Ch4, 8, 9, 13). **(B)** LI between Ch6–8. **(C)** LI between Ch10–13.

According to the analysis of hemispheric dominance, high-OT fathers showed significant left hemispheric dominance in comparison with low-OT fathers (Ch10–13), while low-OT mothers showed significant left hemispheric dominance in comparison with high-OT mothers (Ch6–8, ChL–R). The low-OT mothers also showed significant left hemisphere dominance in comparison with low-OT fathers (Ch6–8, Ch10–13, and ChR–L), while significant hemispheric dominance could not be seen between high-OT mothers and high-OT fathers (Figure 3).

## Discussion

In this study, intermittent separation and reunion of parents and their infants was used to correlate OT and brain activity. It was found that parental OT levels are significantly and positively associated with the activation of the OFC, DLPFC, and FPC during reunion of the subjects with their own infants. The association was stronger among fathers. High-OT fathers were left hemisphere dominant while holding their infants, while the reverse was true among mothers, who were right hemisphere dominant.

Our findings were consistent with previous studies reporting the relationship between OFC and parenting (1). For example, the OFC has been shown to play a major center mediating emphatic and social behaviors, including social cognition and reward-related behavior (32). Holding their own infants naturally attracts adults and motivates the provision of care, which is necessary for infants’ survival and leads to species conservation (33). Holding an infant is also a pleasurable social interaction for a parent, and can be regarded as inherently rewarding (33). The result showing OFC activation can be interpreted as the activation of the reward system evoked by reunion with one’s own infant. On the other hand, the DLPFC and FPC, which are related to executive function (34–36), were activated while holding, possibly because parents considered their infants’ needs based on their facial expression, movements, smells, voice, and body temperature, to decide next actions. Further studies are needed to elucidate the mechanisms on how holding one’s infant evokes parental reward-feelings, consideration, and decision-making.

As expected, significant correlations between parental salivary OT levels and prefrontal activation were observed using fNIRS. This is consistent with previous studies (11, 12) that focused on components of the limbic system, such as the amygdala, NAcc, or hypothalamus and their connectivity with the medial prefrontal cortex (mPFC) and OFC (12). Those findings suggest that OT may directly activate the limbic system and indirectly activates the PFC *via* neural connectivity.

Interestingly, the association between salivary OT levels and brain activation was stronger among fathers. It should, however, be noted that male rats have significantly higher OT receptor densities than females regardless of the estrus phase (37), which may explain our results, even though animal models can’t always be directly extrapolated to humans. We also found differences in the laterality of brain activation between fathers and mothers, which may explain the difference in the association of salivary OT levels and brain activity between them. Left hemispheric dominance was observed among high-OT fathers compared to low-OT fathers, while low-OT mothers showed left hemispheric dominance compared to high-OT mothers. Previous studies on brain lateralization and emotion suggests that the left PFC is associated with approach behaviors and positive emotion, whereas the right PFC is associated with withdrawal behaviors and negative emotion (38). Therefore, because high-OT fathers may experience more positive feelings when they are reunited with their infant than low-OT fathers, their left PFC is more strongly activated than low-OT fathers. On the other hand, high-OT mothers may have more negative feelings than low-OT mothers, although we did not assess the feeling *per se* directly. A previous study reported that maternal plasma OT positively correlated with the proportion of time mothers spent engaging in affectionate contact, i.e., kissing, patting, or light poking (7). It possibly suggests that high-OT mothers may feel more stressed and responsible to hold infants in a physically straining way than low-OT mothers, and that the strain might evoke negative feelings among high-OT mothers. Further, there might be a difference on the brain activity of decision making after reunion. Parents need to decide how to behave toward infants after reunion, and in this process, fathers may tend to take approach behaviors, which was shown as high activity of left PFC, whereas mothers may tend to take withdrawal approach, which was shown as high activity of right PFC, possibly because holding infant can be novel stimulus for fathers, but not for mothers. To establish a link between salivary OT and brain activity in mothers a more in-depth investigation needs to be considered.

Most studies have challenges and in the context of this study, the following limitations should be noted. First, we had no comparative experiments with non-blood-related infants, and no control for subjects such as unmarried adults or adults without children. These factors are a cause for concern. Without the abovementioned control groups, the observed brain activation may have been human adults’ innate reactions and not purely parental behavior. Second, although the study was closer to taking real-world measurements than previous studies that used simply pictures or videos of their infants, subjects could not behave freely and were restricted to a seated position and instructed not to make exaggerated movements. Third, a large number of mothers (28.6%) were excluded due to artifacts during fNIRS. Probes attached to mothers were seemingly looser than those attached to fathers, because of larger volumes of hair. Moreover, spatial resolution of fNIRS is inferior to fMRI, thus the brain regions, such as OFC, DLPFC, and FPC associated with OT level in our study might not be perfectly accurate. Fourth, saliva OT may not be the same as cerebrospinal OT concentration, which would reflect the most accurate OT levels in the brain. Although saliva OT would be the least accurate sample, taking saliva is the best way for the purpose of this study in terms of invasion and stress which would affect OT levels. Significant correlation of OT levels between plasma and saliva in the parents has been already shown (17). A further, larger study is needed to replicate our findings.

Based on this study, we could provide rationale to investigate the impact of elevating OT level for appropriate parenting, especially among fathers. To date, some clinical trials of nasal OT inhalation have been conducted for patients with autism spectrum disorder, schizophrenia, social anxiety, and post-traumatic stress disorder (39). That is, randomized controlled trial of elevating OT level, using direct method [e.g., nasal spray of OT (19)] or indirect method [e.g., touching or hugging (20)], among parents at risk of poor parenting is warranted.

In conclusion, parental OT levels were significantly positively associated with activation of the OFC, DLPFC, and FPC during reunion with their own infants, with a higher correlation among fathers. Moreover, fathers with higher OT levels showed left hemisphere dominant neural activation while holding their infants, although the same is not true for mothers. Further investigations are warranted to elucidate this physiological difference between fathers and mothers.

## Ethics Statement

This study was approved by the ethics committee of the National Center for Child Health and Development, and all participants signed informed consent forms.

## Author Contributions

TF and JI conceived study design, JI implemented, collected, analyzed data, and wrote first draft, YM and TY analyzed data, and TF, YM, and HO interpreted results and revised manuscript, TF and HO finalized the manuscript.

## Conflict of Interest Statement

The authors declared no potential conflicts of interest with respect to the research, authorship, and/or publication of this article.

## Abbreviations

OT, oxytocin; fNIRS, functional near-infrared spectroscopy; PFC, prefrontal cortex; OFC, orbitofrontal cortex; DLPFC, dorsolateral prefrontal cortex; FPC, frontopolar cortex; NAcc, nucleus accumbens; fMRI, functional magnetic resonance imaging.
